# Fomiroid A, a Novel Compound from the Mushroom *Fomitopsis nigra*, Inhibits NPC1L1-Mediated Cholesterol Uptake via a Mode of Action Distinct from That of Ezetimibe

**DOI:** 10.1371/journal.pone.0116162

**Published:** 2014-12-31

**Authors:** Tomohiro Chiba, Tsuyoshi Sakurada, Rie Watanabe, Kohji Yamaguchi, Yasuhisa Kimura, Noriyuki Kioka, Hirokazu Kawagishi, Michinori Matsuo, Kazumitsu Ueda

**Affiliations:** 1 FANCL Research Institute, FANCL Corporation, 12-13 Kamishinano, Totsuka-ku, Kanagawa 244-0806, Japan; 2 Division of Applied Life Sciences, Graduate School of Agriculture, Kyoto University, Kitashirakawa Oiwake-cho, Sakyo-ku, Kyoto 606-8502, Japan; 3 Institute for Integrated Cell-Material Sciences (iCeMS), Kyoto University, Kitashirakawa Oiwake-cho, Sakyo-ku, Kyoto 606-8502, Japan; 4 Graduate School of Science and Technology, Shizuoka University, 836 Ohya, Suruga-ku, Shizuoka 422-8529, Japan; 5 Research Institute of Green Science and Technology, Shizuoka University, 836 Ohya, Suruga-ku, Shizuoka 422-8529, Japan; 6 Graduate School of Agriculture, Shizuoka University, 836 Ohya, Suruga-ku, Shizuoka 422-8529, Japan; 7 Department of Food and Nutrition, Kyoto Women's University, Kitahiyoshi-cho, Imakumano, Higashiyama-ku, Kyoto 605-8501, Japan; Nihon University School of Medicine, Japan

## Abstract

Hypercholesterolemia is one of the key risk factors for coronary heart disease, a major cause of death in developed countries. Suppression of NPC1L1-mediated dietary and biliary cholesterol absorption is predicted to be one of the most effective ways to reduce the risk of hypercholesterolemia. In a screen for natural products that inhibit ezetimibe glucuronide binding to NPC1L1, we found a novel compound, fomiroid A, in extracts of the mushroom *Fomitopsis nigra*. Fomiroid A is a lanosterone derivative with molecular formula C_30_H_48_O_3_. Fomiroid A inhibited ezetimibe glucuronide binding to NPC1L1, and dose-dependently prevented NPC1L1-mediated cholesterol uptake and formation of esterified cholesterol in NPC1L1-expressing Caco2 cells. Fomiroid A exhibited a pharmacological chaperone activity that corrected trafficking defects of the L1072T/L1168I mutant of NPC1L1. Because ezetimibe does not have such an activity, the binding site and mode of action of fomiroid A are likely to be distinct from those of ezetimibe.

## Introduction

Cholesterol is an important membrane component, as well as a precursor for biosynthesis of steroid hormones, biliary acid, and vitamin D. However, excess accumulation of cholesterol in artery wall causes coronary heart disease [1], [2]. Therefore, the free cholesterol level in the body is tightly controlled by regulation of *de novo* synthesis, dietary and biliary cholesterol absorption, biliary excretion, and storage as cholesterol esters. Various drugs used for treatment of hypercholesterolemia target these mechanisms. One such drug, ezetimibe, targets dietary and biliary cholesterol absorption [3]–[6].

Ezetimibe binds directly to Niemann-Pick C1-like 1 (NPC1L1), which is highly expressed at apical surface of enterocytes and biliary canaliculi in humans; inhibition of this protein prevents cholesterol absorption from the small intestine and reabsorption from biliary canaliculi [7]–[13]. NPC1L1 is a homologue of NPC1, which is involved in cholesterol transfer in the lysosome, and consists of 13 putative transmembrane α helices (TMs); the conserved sterol-sensing domain (SSD) is present in TM4–8 [14]. Cholesterol and oxysterols bind directly to the amino-terminal domain (NTD) [15], triggering clathrin/AP2–mediated endocytosis of NPC1L1 and leading to cholesterol uptake [16]. Ezetimibe binds to the second extracellular domain between TM2 and TM3 [12], and blocks cholesterol-induced endocytosis [17]. Several steroidal and non-steroidal compounds have pharmacological chaperone activities that correct trafficking defects of NPC1L1 mutants. Recently, Karaki *et al.* reported that such compounds bind to sites distinct from both the NTD and the ezetimibe-binding site, suggesting the existence of a second sterol-binding site in NPC1L1 [18], [19].

In a natural-product screen using plant and mushroom extracts, we identified a novel compound in extracts of the mushroom *Fomitopsis nigra* that binds to NPC1L1 and prevents NPC1L1-mediated cholesterol uptake. Because this compound, fomiroid A, exhibits a pharmacological chaperone activity that ezetimibe lacks, we predict that its binding site and the mode of action are distinct from those of ezetimibe.

## Materials and Methods

### Antibodies and reagents

[1,2-^3^H(N)]-cholesterol was purchased from PerkinElmer. [^3^H(G)] Ezetimibe β-D-glucuronide ([^3^H]EZG) was purchased from American Radiolabeled Chemicals. Ezetimibe was obtained from Toronto Research Chemicals. CellMask Orange plasma membrane stain was obtained from Life Technologies.

The following antibodies were used in this study: mouse monoclonal anti-HA (F-7, sc-7392, Santa Cruz Biotechnology), anti-GFP (B-2, sc-9996, Santa Cruz Biotechnology), anti-vinculin (V9131, Sigma-Aldrich), rabbit polyclonal anti-NPC1L1 (HPA018105, Sigma-Aldrich), horseradish peroxidase (HRP)-rabbit anti–mouse IgG (H+L) conjugate (81-6720, Life Technologies), HRP-goat anti-rabbit IgG (H+L) conjugate (81-6120, Life Technologies), and Alexa Fluor 633 goat anti–mouse IgG (H+L) conjugate (A-21052, Life Technologies).

Other chemicals were purchased from Sigma-Aldrich, Wako Pure Chemical Industries, or Nacalai Tesque.

### Isolation and characterization

Fresh fruiting bodies of *Fomitopsis nigra* (42 kg) were successively extracted with ethanol (168 L) and acetone (42 L). After the solutions were combined and concentrated under reduced pressure, the concentrate was partitioned between *n*-hexane and H_2_O. The *n*-hexane-soluble fraction (28.3 g) was further divided into hexane-soluble and acetonitrile (MeCN)/H_2_O (8∶2)-soluble fractions. The acetonitrile-soluble fraction (13.6 g) was further fractionated by C18 reverse-phase chromatography (ODS-A, pore size 6 nm, particle size 75 µm, YMC, MeCN/H_2_O 75∶25, acetone) to yield eight fractions (fractions 1 to 8). Part of fraction 2 (73 mg) was further separated by reverse-phase HPLC (Develosil XG C30-M5, Nomura Chemical, MeCN/H_2_O 6∶4) to yield 10 fractions (2–1 to 2–10). An active compound was purified from fraction 2–6 by reverse-phase HPLC (Develosil XG C30-M5, MeCN/H_2_O 6∶4, MeCN/H_2_O including 0.1% (v/v) formic acid 6∶4).^ 1^H NMR spectra (one- and two-dimensional) were recorded on a Bruker AVANCE 500 NMR at 500 MHz with a CryoProbe in methanol- *d*_4_ and pyridine- *d*_5_, using tetramethylsilane as the internal standard, and ^13^C NMR spectra were recorded on the same instrument at 125 MHz. HRESIMS was measured on a Waters Synapt G2-S mass spectrometer. A Perkine-Elmer Spectrum 400 FT-IR/NIR spectrometer equipped with a universal ATR sampling accessory was used to record the IR spectra, and the specific rotation values were measured on a JASCO DIP1000 polarimeter. Active compound: [α]_D_ +79 (*c* 0.35, 24°C, CHCl_3_); IR (neat): 3433, 2938, 1704, 1375, 1150, 1052, 1014 cm^−1^; ^1^H and ^13^C NMR, see Table 1.

**Table 1:** ^1^H (500 MHz) and ^13^C (125 MHz) NMR data.

	fomiroid A (CD_3_OD)			fomiroid A (C_5_D_5_N)		[Ref. 20] (C_5_D_5_N)	
No	δ_H_ (multiplicity, *J* in Hz)	δ_c_	HMBC correlation	δ_H_ (multiplicity, *J* in Hz)	δ_c_	δ_H_ (multiplicity, *J* in Hz)	δ_c_
1	1.63 (*m*)	37.2			36.2		36.7
	2.05 (*m*)		C-3, C-5, C-19				
2	2.39 (*ddd*, 3.6, 7.0, 15.9)	35.5	C-1, C-3, C-4		34.8		35.0
	2.61 (*ddd*, 7.2 10.9, 15.9)		C-1, C-3				
3		220.5			216.4		216.2
4		48.5			47.4		47.6
5	1.63 (*m*)	52.6			51.2		51.5
6	1.63 (*m*)	20.6			18.7		19.0
7	2.23 (*m*)	28.1	C-5, C-6, C-8		27.5		27.6
8		136.4			133.7		133.6
9		135.0			135.5		135.2
10		38.3			37.2		37.5
11	2.10 (*m*)	21.9	C-9, C-12, C-13		21.2		21.6
12	1.49 (*m*)	32.2	C-9, C-11, C-13, C-14, C-18		30.7		31.0
	1.91 (*m*)		C-11, C-13, C-14, C-18				
13		45.9			44.9		45.0
14		53.2			52.5		52.8
15	4.20 (*dd*, 5.9, 9.2)	73.8	C-8, C-14, C-16, C-17, C-30	4.57 (*dd*, 6.0, 8.7)	72.3	4.60 (*t*, 7.2)	72.7
16	1.76 (*m*)	39.6	C-15, C-17		39.7		40.1
	1.99 (*m*)						
17	1.98 (*m*)	44.8			44.2		44.4
18	0.82 (*s*)	17.0	C-12, C-13, C-14, C-30	0.92 (*s*)	16.8	0.98 (*s*)	17.3
19	1.14 (*s*)	19.0	C-5, C-9, C-10	1.02 (*s*)	19.8	1.06 (*s*)	20.1
20	1.65 (*m*)	41.1			40.3		44.0
21	4.03 (*dd*, 5.1, 11.3)	66.4	C-17, C-20, C-22	4.31 (*dd*, 5.9, 10.8)	65.6	3.94 (*dd*, 5.3, 10.6)	62.2
	4.29 (*dd*, 2.3, 11.3)		C-17, C-20, C-22	4.63 (*dd,* 2.1, 10.8)		4.10 (*br*, 10.6)	
22	1.35 (*m*)	31.2	C-17, C-20, C-21, C-23, C-24		31.4		31.9
	1.46 (*m*)		C-17, C-20, C-21, C-23, C-24				
23	1.98 (*m*)	25.8	C-22, C-24, C-25		25.0		25.9
24	5.10 (*t*, 7.0)	125.7	C-22, C-23, C-26, C-27	5.24 (*t*, 6.8)	125.4	5.29 (*m*)	126.1
25		132.4			131.3		130.8
26	1.67 (*s*)^a^	25.9	C-24, C-25, C-26	1.63 (*s*)	25.8	1.60 (*s*)	26.1
27	1.62 (*s*)^a^	17.8	C-24, C-25, C-27	1.67 (*s*)	17.7	1.66 (*s*)	18.1
28	1.08 (*s*)^b^	26.8	C-3, C-4, C-5, C-29	1.14 (*s*)	26.4	1.13 (*s*)	26.7
29	1.07 (*s*)^b^	21.8	C-3, C-4, C-5, C-28	1.09 (*s*)	21.3	1.07 (*s*)	21.6
30	0.96 (*s*)	17.8	C-8, C-13, C-14, C-15, C-18	1.30 (*s*)	18.0	1.33 (*s*)	18.4

a, b interchangeable.

### Cell culture

Human embryonic kidney 293 cells (HEK293, CRL-1573), human embryonic kidney 293T cells (CRL-3216), and human colon carcinoma Caco2 cells (HTB-37) were purchased from the American Type Culture Collection. Cells were maintained in Dulbecco’s modified Eagle’s medium (DMEM, Life Technologies) containing 10% heat-inactivated fetal bovine serum (FBS) at 37°C in 5% CO_2_.

### DNA constructs, transfection, and stably expressing cell lines

The cDNA encoding rat NPC1L1 (rNPC1L1) (GenBank AY437867) was generated by PCR amplification from rat jejunum single-strand cDNA (GenoStaff) and cloned into pCR4 Blunt TOPO (Life Technologies). The rNPC1L1 cDNA from this construct was inserted into the expression vector pcDNA3.1 (Life Technologies) and pCDH-CMV-MCS-EF1-Puro Lentivector (System Biosciences).

The cDNA encoding the human NPC1L1 (hNPC1L1) (GenBank AY437865) was generated by PCR amplification from a human liver cDNA library (Takara Bio) and cloned into pCR2.1 (Life Technologies). The hNPC1L1 cDNA was modified by inserting enhanced green fluorescent protein (EGFP) sequence at the C terminus, and then ligated into pcDNA3.1. Site-directed mutagenesis was performed by a PCR-based strategy using iProof high-fidelity DNA polymerase (Bio-Rad Laboratories). The influenza virus hemagglutinin (HA) tag was inserted between S986 and L987 of hNPC1L1.

The pcDNA3.1 constructs encoding rNPC1L1, hNPC1L1-EGFP, hNPC1L1^L1072T/L1168I^-EGFP, HA-hNPC1L1-EGFP, or HA-hNPC1L1^L1072T/L1168I^-EGFP were transfected into HEK293 cells using FuGENE HD (Promega). Cells were selected by culturing in the presence of 1 mg/ml G418 sulfate (Wako Pure Chemical Industries). For FACS analysis, HEK293 cells stably expressing HA-hNPC1L1^L1072T/L1168I^-EGFP were cloned by limiting dilution.

### Membrane preparation

HEK293 cells stably expressing rNPC1L1 were treated with 4 mM sodium butyrate (Sigma-Aldrich) for 24 h, and then used for membrane preparation essentially according the methods of Garcia-Calvo *et al.* [10]. Briefly, cells suspended in buffer containing 20 mM HEPES/Tris buffer (pH 7.4), 8% sucrose, and proteinase inhibitors (complete EDTA-free; Roche Applied Science) were disrupted with a probe sonicator (Misonix) on ice, and then centrifuged at 1,500×*g* for 10 min at 4°C to remove unbroken cells and nuclei. The supernatants were centrifuged at 125,000×*g* for 3 h at 4°C, and the resultant pellets were resuspended in ice-cold buffer containing 20 mM HEPES/Tris buffer (pH 7.4), 160 mM NaCl, and 5% glycerol. Samples were stored at −80°C prior to use.

### Competitive binding assay

Binding of [^3^H]EZG to membranes from HEK293 cells stably expressing rNPC1L1 (HEK/rNPC1L1) was performed as reported previously [10] with minor modifications. Briefly, reactions were carried out at room temperature for 1 h in 96-well microplates, in binding buffer containing 5 mM HEPES (pH 7.4), 5.5 mM glucose, 117 mM NaCl, 5.4 mM KCl, 0.03% sodium taurocholate, and 0.05% digitonin. [^3^H]EZG concentration was 25 nM, membrane protein concentration was 37.5 µg/assay, test compound concentrations were 23 nM–4 mM, and total assay volume was 30 µl. Fomiroid A and ezetimibe were dissolved in ethanol/dimethyl sulfoxide (EtOH/DMSO 1∶1) solution. Fomiroid A was soluble at 80 mM in this solution. The final concentration of EtOH/DMSO in the reaction mixture was 2.5/2.5%. Nonspecific binding was measured in the presence of 100 µM ezetimibe. At end of the incubation period, samples were transferred to Unifilter-96 GF/C plates (PerkinElmer) using a Unifilter-96 Harvester (PerkinElmer). The plates were washed several times with Milli-Q water (Merck Millipore) and dried. Fifteen microliters of Microscint-20 (PerkinElmer) was added to each filter, and radioactivity was measured on a TopCount (PerkinElmer).

IC_50_ values were estimated by fitting the default one-site or two-site competition models to the data after best-fit comparison of the two corresponding equations in the GraphPad Prism software, version 4.0 (*F* test, *p*<0.05).

### Western blotting

Cells were washed with ice-cold PBS and lysed at 4°C in RIPA buffer containing 50 mM Tris-HCl (pH 7.6), 150 mM NaCl, 1% Triton X-100, 0.5% sodium deoxycholate, 0.1% sodium dodecyl sulfate (SDS), and proteinase inhibitors (Nacalai Tesque). Protein concentration was determined using the BCA Assay kit (Pierce Biotechnology). Samples were separated by SDS-PAGE (7.5%), and the resolved proteins were transferred to a PVDF membrane (GE Healthcare). Proteins on PVDF membranes were treated with anti-GFP (1∶3000 dilution), anti-NPC1L1 (1∶1500 dilution), or anti-vinculin (1∶50000 dilution) primary antibodies, and then incubated with HRP-labeled rabbit anti–mouse IgG or HRP-labeled goat anti–rabbit IgG secondary antibodies (1∶10000). Chemiluminescence images were captured on an LAS-4000 mini (Fujifilm), and densitometry of non-saturated band images was performed using the Multi Gauge software (Version 3.2, Fujifilm).

### Confocal imaging of NPC1L1 and Plasma membrane

HEK293 cells stably expressing hNPC1L1-EGFP or hNPC1L1^L1072T/L1168I^-EGFP were seeded onto 35-mm poly-D-lysine–coated glass-bottomed dishes. The next day, cells were treated with test compound. After 24 h, the cells were stained with 2.5 µg/ml CellMask Orange plasma membrane stain in Hank's Balanced Salt Solution (HBSS) at 37°C for 5 min, followed by three washes with HBSS. Live cells in fresh HBSS were then visualized by confocal laser microscopy (FV1000, Olympus).

### FACS analysis

HEK293 cells stably expressing HA-hNPC1L1^L1072T/L1168I^-EGFP were seeded onto 12-well plates at a density of 5×10^5^ cells/well. Next day, the cells were treated with test compound for 24 h, and then harvested with Accutase (Innovative Cell Technologies) and suspended in HBSS containing 1% bovine serum albumin (BSA) for 15 min at 4°C. After centrifugation, HBSS containing 1% BSA, anti-HA antibody (1∶250 dilution), and Alexa Fluor 633–conjugated anti–mouse IgG antibody (1∶500 dilution) were added to the cell pellets and mixed. The cell suspensions containing antibodies were incubated for 30 min at room temperature. After three washes with HBSS, the cells were resuspended in HBSS and immediately subjected to FACS analysis (FACSCalibur, BD Biosciences).

### Production of lentiviral stock and Caco2 cell infection

293T cells were grown on 100-mm dishes for 24 h prior to transfection. pCDH/rNPC1L1 (or pCDH vector), pMD2.G (Addgene), and psPAX2 (Addgene) were transfected using Lipofectamine LTX (Life Technologies). Transfected cells were cultured for 96 h and the supernatant containing lentiviral particles was centrifuged at 2,000×*g* for 10 min, and then passed through a 0.45-µm filter. Caco2 cells were seeded onto 6-well plates at 2.5×10^5^ cells/well. After 24 h, medium was replaced with 0.5 ml of conditioned lentiviral medium, 1.5 ml of DMEM containing 10% FBS, and 5 µg/ml Polybrene (Santa Cruz Biotechnology). Infected cells were selected by culture in the presence of 5 µg/ml puromycin (Life Technologies).

### Cholesterol uptake and esterification

Caco2/mock or Caco2/rNPC1L1 cells were seeded onto 12-well plates at a density of 5×10^5^ cells/well in DMEM containing 10% FBS, 100 units/ml penicillin, and 100 µg/ml streptomycin, and then cultured for 10–14 days to allow differentiation.

To study [^3^H]cholesterol uptake, cells were incubated with a micellar solution containing 2 mM sodium taurocholate, 50 µM phosphatidylcholine, 1 µM cholesterol, 1 µCi/ml [^3^H]cholesterol, and the indicated concentrations of ezetimibe or test compound for 1 h at 37°C. After three washes with PBS(+), cells were treated overnight at room temperature with 0.2 N NaOH containing 0.1% lithium dodecyl sulfate (LDS), and radioactivity was counted in a liquid scintillation counter (LSC-5100, ALOKA). Total protein concentrations were determined using the BCA assay kit.

To measure [^3^H]esterified cholesterol, cells were incubated at 37°C with a micellar solution containing 5 mM sodium taurocholate, 500 µM oleate, 10 µM cholesterol, 1 µCi/ml [^3^H]cholesterol, and the indicated concentrations of ezetimibe or test compound. After 1 h, the solution was replaced with DMEM containing Insulin-Transferrin-Selenium (ITS) (Life Technologies). To produce esterified cholesterol, cells were incubated for 8 h at 37°C. After three washes with PBS(+), lipids were extracted twice with 500 µl of hexane-isopropanol (3∶2), which was transferred to glass tubes. After evaporation, the lipids were resuspended in chloroform:methanol (2∶1). Esterified cholesterol was separated from other lipids by silica-based thin-layer chromatography (TLC) (Merck Millipore) using hexane/diethyl ether/acetate (6∶4∶0.1). After the staining of TLC plates with iodine vapor, esterified cholesterol was scraped from the plate, and radioactivity was counted. The lipid-extracted cells were treated overnight at room temperature with 0.2 N NaOH containing 0.1% LDS, and then used for protein determination.

### Statistical analysis

All data are presented as means ± S.E. Statistical analyses were performed by one-way ANOVA followed by using Dunnett’s test. *p*<0.05 was considered to represent a statistically significant difference.

## Results

### Structure determination of fomiroid A from *Fomitopsis nigra*

To obtain compound(s) that modify the function of NPC1L1, we employed a competitive-binding assay using membranes from rNPC1L1-expressing cells and [^3^H]EZG to screen natural products such as plant and mushroom extracts. The extract of the mushroom *Fomitopsis nigra* exhibited the desired activity. Next, we examined whether the extract exhibited cholesterol-lowering effects on cholesterol diet-fed rats, and found that the acetonitrile-soluble fraction reduced plasma cholesterol levels after oral administration at a dose of 500 mg/kg/day (data not shown). Then, we further fractionated it by repeated column chromatography, guided by the results of the competitive-binding assay. Ultimately, we isolated the active compound.

**Figure 1:**
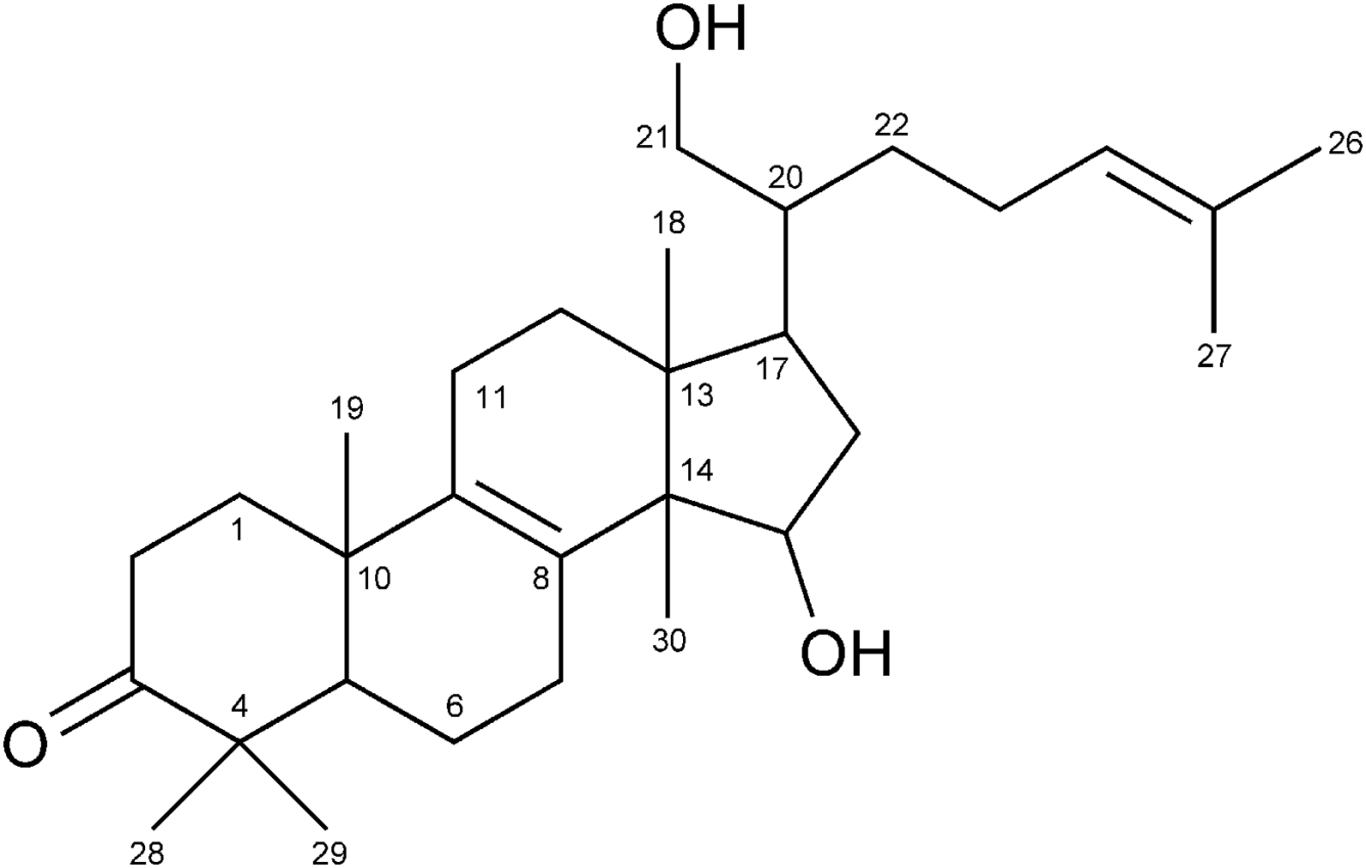
Structure of fomiroid A.

The active compound was obtained as a colorless oil. Its molecular formula was determined as C_30_H_48_O_3_ by HR-ESI-MS (*m/z* 455.3529 [M-H]^−^; calculated for C_30_H_47_O_3_: 455.3525), indicating the presence of seven degrees of unsaturation in the molecule. The structure of the active compound was elucidated by interpretation of NMR spectra including DEPT, COSY, HMQC, and HMBC. A DEPT experiment indicated the presence of seven methyls, ten methylenes, five methines, and eight quaternary carbons. Together, the molecular formula, the degrees of unsaturation, ^13^C NMR data (δ_C_ 66.4, 73.8, 125.7, 132.4, 135.0, 136.4, 220.5), and the DEPT data indicated the presence of four rings, two double bonds, two hydroxyl groups, and a carbonyl group in the molecule. Overall, these features suggested that the compound is a lanosterone derivative. The COSY spectrum revealed connectivities of C-1 to C-2, C-6 to C-7, C-11 to C-12, C-15 to C-16, C-20 to C-21, C-23 to C-24, and C-24 to C-26 and C-27. HMBC correlations revealed two double bonds at C-8 and C-24, two hydroxyl groups attached at C-15 and C-21, and a ketone group attached at C-3. The complete assignment of the protons and carbons of NMR and HMBC correlations are summarized in Table 1. Based on these data, the structure of the compound was determined to be 15, 21-dihydroxylanosta-8, 24-dien-3-one (Fig. 1). Previously, a patent was filed for use of a compound with the same planar structure, isolated from the mushroom *Fomitopsis pinicola* [20], as an inhibitor against cyclooxygenase and lipoxygenase. However, the ^13^C NMR chemical shifts of the compounds were distinct, especially those at C-20 and C-21, suggesting that the active compound in this study is a novel diastereomer at the side chain of the previously reported compound.

### Effect of fomiroid A on [^3^H]ezetimibe-glucuronide binding to NPC1L1

**Figure 2:**
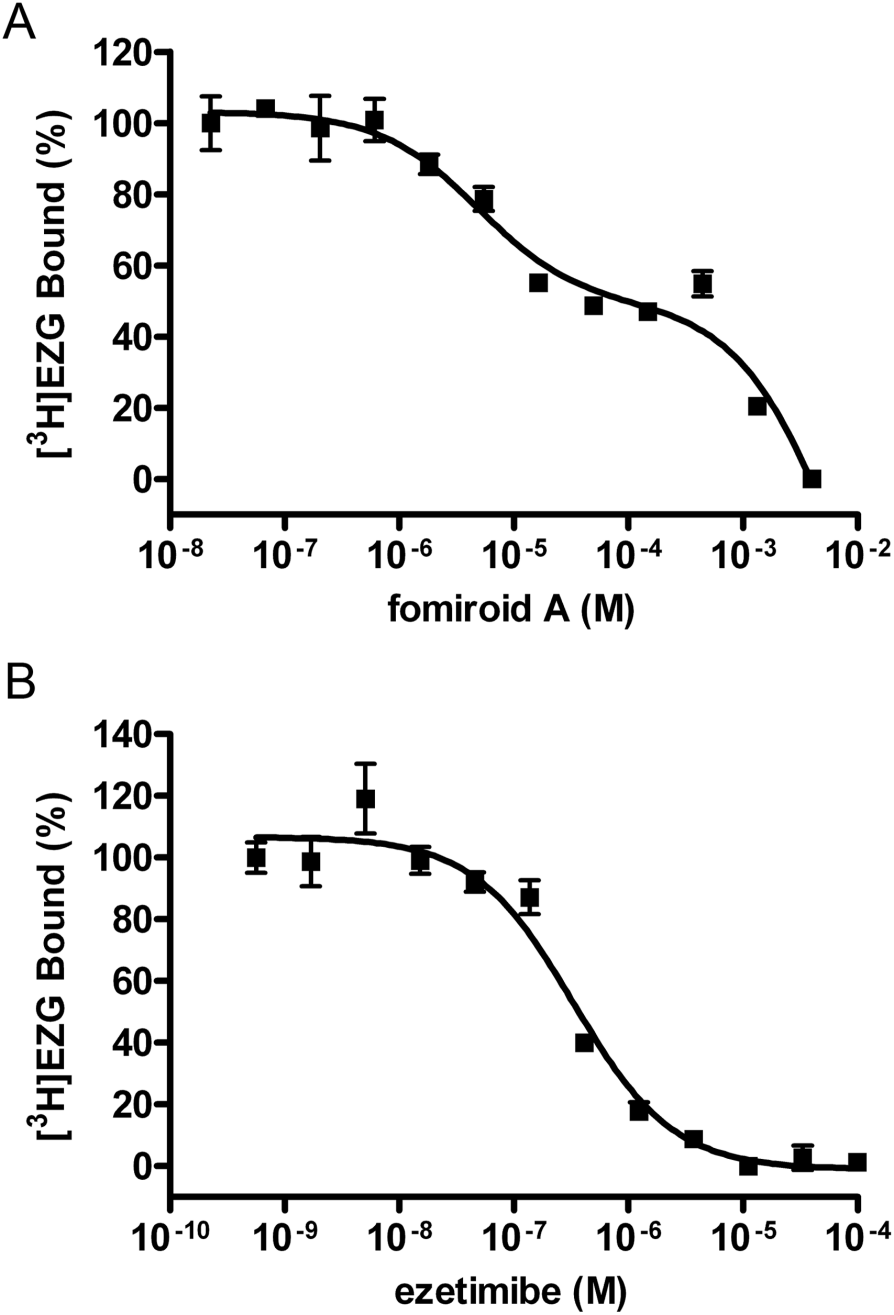
Inhibition of specific [^3^H]ezetimibe glucuronide binding to NPC1L1. A–B, Membranes (37.5 µg protein) from HEK293/rNPC1L1 were incubated for 1 h at room temperature with 25 nM [^3^H]EZG in the presence of increasing concentrations of unlabeled fomiroid A (A) or ezetimibe (B). Nonspecific binding was measured with 100 µM ezetimibe. IC_50_ values were estimated by fitting the data to the default one-site (ezetimibe treatment) or two-site (fomiroid A treatment) competition models using the Prism software. Values represent means ± S.E. (n = 4).

Concentration-dependent inhibition of [^3^H]EZG binding by fomiroid A and ezetimibe is demonstrated in Fig. 2. Fomiroid A inhibited [^3^H]EZG binding apparently in a biphasic manner, whereas ezetimibe exhibited monophasic competition. Statistical analysis suggested that a two-site model for fomiroid A binding was preferable to a one-site model. The IC_50_ values were calculated to be 4.83±0.2 µM for the high-affinity site and 1.45±0.1 mM for the low-affinity site. By contrast, the IC_50_ value of ezetimibe was calculated to be 332±4.2 nM.

### Activity of fomiroid A as a pharmacological chaperone

**Figure 3:**
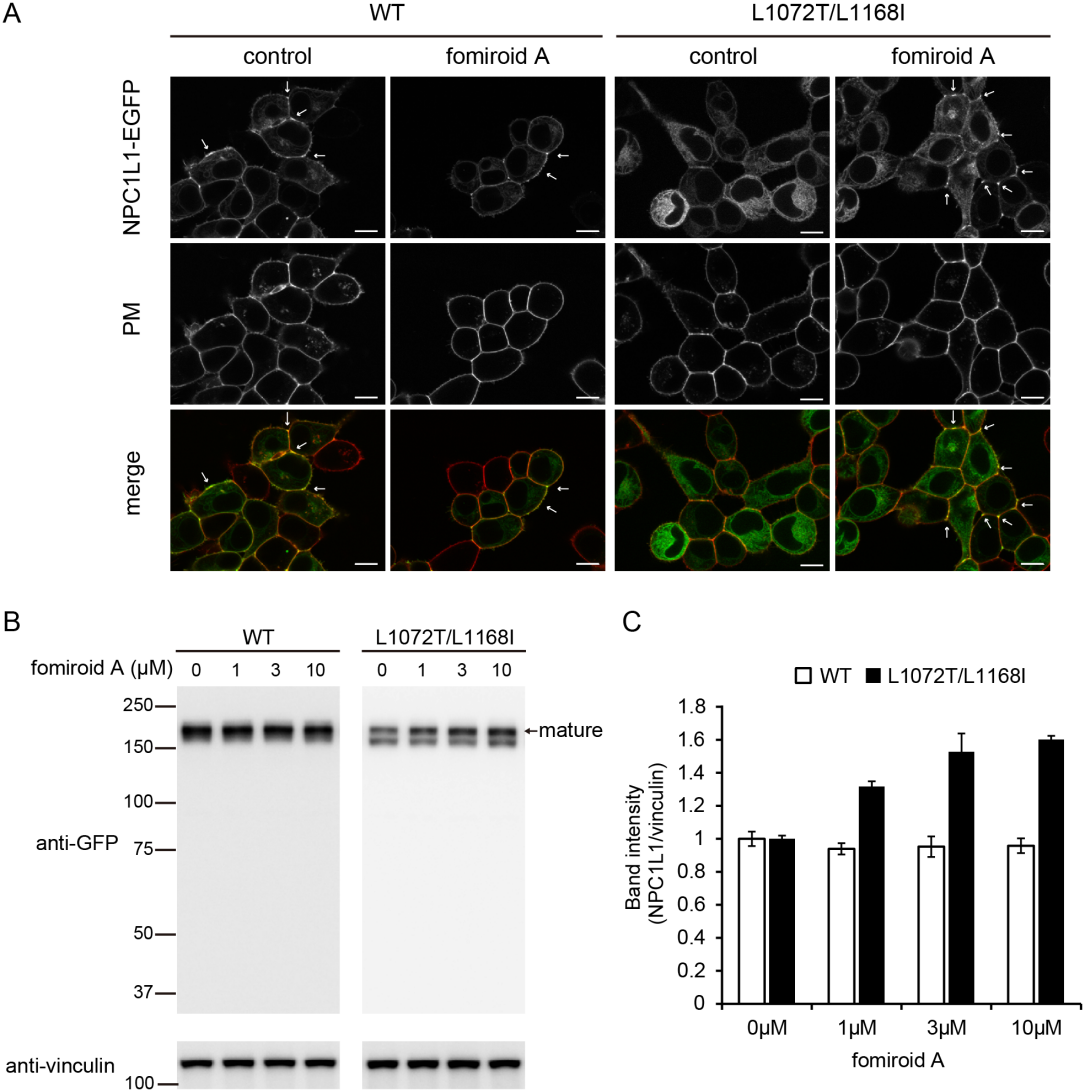
Rescue of mislocalized NPC1L1^L1072T/L1168I^. A, HEK293 cells stably expressing hNPC1L1-EGFP (WT) or hNPC1L1^L1072T/L1168I^-EGFP (L1072T/L1168I) were incubated in the absence or presence of 10 µM fomiroid A for 24 h, and then plasma membranes (PM) were stained with CellMask Orange. The arrows show the rescue of mislocalized L1072T/L1168I mutant by fomiroid A treatment. Scale bar, 10 µm. B, WT or L1072T/L1168I mutant cells were treated with the indicated concentrations of fomiroid A for 24 h. The amount of NPC1L1 was determined using anti-GFP antibody. Vinculin was used as a loading control. C, Intensities of bands representing mature NPC1L1. Values represent means ± S.E. (n = 3). *p<0.05 compared with untreated mutant cells.

Several steroidal and non-steroidal compounds, which bind to NPC1L1, have pharmacological chaperone activities that correct trafficking defects of NPC1L1 mutants. Recently, it was reported that such compounds bind to sites distinct from both the NTD and the ezetimibe-binding site [18], [19]. To determine whether fomiroid A directly binds to hNPC1L1, we analyzed the pharmacological chaperone activity of this compound on trafficking of the L1072T/L1168I mutant to the plasma membrane. As shown in Fig. 3A, when expressed in HEK293 cells, the L1072T/L1168I mutant primarily localized at endoplasmic reticulum and barely co-localized with the plasma-membrane marker, whereas the wild-type (WT) protein primarily localized to the plasma membrane and co-localized extensively with its marker. The addition of fomiroid A increased the localization of L1072T/L1168I mutant to the plasma membrane, but did not affect the subcellular localization of WT.

To confirm that the plasma-membrane localization of the L1072T/L1168I mutant was increased by fomiroid A, we analyzed the maturation of hNPC1L1 by western blotting. As shown in Fig. 3B, the expression pattern of WT was not affected by fomiroid A treatment. On the other hand, the L1072T/L1168I mutant was expressed as two forms, mature (upper) and immature (lower), and the intensity of the mature band was significantly increased by fomiroid A treatment in a dose-dependent manner (Fig. 3C).

**Figure 4:**
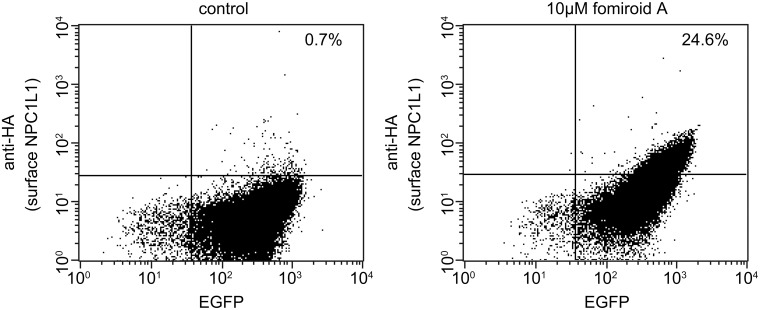
Quantitation of cell-surface expression of HA-tagged NPC1L1^L1072T/L1168I^ by FACS analysis. HEK293 cells stably expressing HA-hNPC1L1^L1072T/L1168I^-EGFP (HA-L1072T/L1168I) were incubated in the absence or presence of 10 µM fomiroid A for 24 h. Cell-surface expression of HA-L1072T/L1168I mutant was detected by staining with anti-HA and Alexa Fluor 633–conjugated anti–mouse IgG antibodies, and then quantitated by FACS analysis. The percentages in the upper right regions of the panels indicate the proportion of cells double-positive for anti-HA antibody and EGFP.

In addition, we measured the amount of NPC1L1 on the cell surface by FACS analysis. For these experiments, we established cells stably expressing the L1072T/L1168I mutant, in which the HA tag was introduced between positions 986 and 987 in the fifth extracellular domain (HA-L1072T/L1168I) [21], [22]. Because [^3^H]EZG binding was not affected (S1 Fig.) and fomiroid A facilitated the localization of HA-L1072T/L1168I mutant to the plasma membrane (S2 Fig.), we inferred that insertion of the HA tag had a minimal effect on the structure and function of NPC1L1. The fraction of cells double-positive for anti-HA antibody and EGFP increased from 0.7% to 24.6% upon treatment of fomiroid A, indicating that the localization of the HA-L1072T/L1168I mutant on the cell surface was promoted by fomiroid A treatment (Fig. 4). Together, these results suggest that fomiroid A acts as a pharmacological chaperone by binding to NPC1L1.

### Effects of fomiroid A on cholesterol uptake and esterification

**Figure 5:**
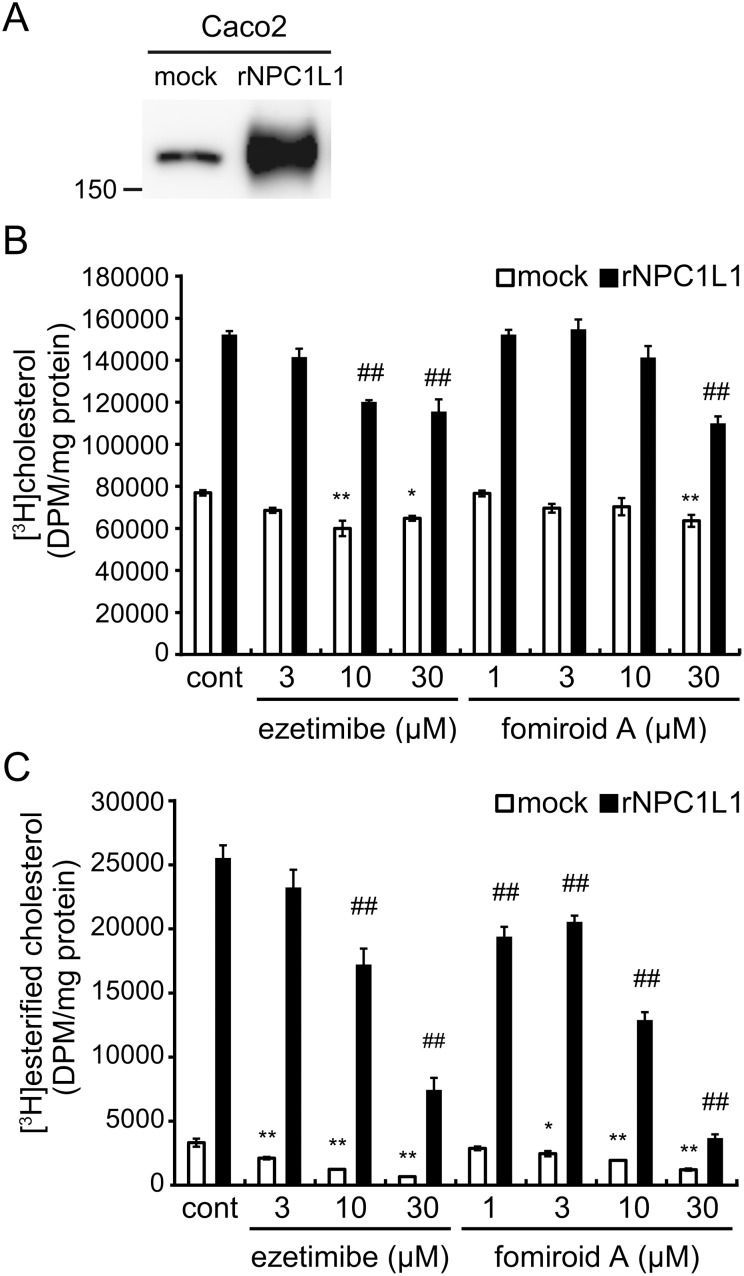
Inhibitory effects of fomiroid A on cholesterol uptake and esterification in Caco2/mock and Caco2/rNPC1L1 cells. A, Expression of NPC1L1 was determined by western-blot analysis using anti-NPC1L1 antibody. B, Differentiated Caco2/mock and Caco2/rNPC1L1 cells were incubated for 1 h at 37°C with a micellar solution containing 2 mM sodium taurocholate, 50 µM phosphatidylcholine, 1 µM cholesterol, 1 µCi/ml [^3^H]cholesterol, and the indicated concentrations of ezetimibe or fomiroid A. Radioactivity of [^3^H]cholesterol was counted in a liquid scintillation counter. C, Differentiated Caco2/mock and Caco2/rNPC1L1 cells were incubated at 37°C with a micellar solution containing 5 mM sodium taurocholate, 500 µM oleate, 10 µM cholesterol, 1 µCi/ml [^3^H]cholesterol, and the indicated concentrations of ezetimibe or fomiroid A; after 1 h, the micellar solution was replaced with DMEM containing Insulin-Transferrin-Selenium. The cells were incubated for 8 h at 37°C. The lipids extracted by organic solvent were separated with TLC, and the radioactivity of [^3^H]esterified cholesterol was counted by a liquid scintillation counter. Values represent the means ± S.E. (n = 3). *p<0.05, **p<0.01 compared with control Caco2/mock cells, ^#^ *p*<0.05, ^##^ *p*<0.01 compared with control Caco2/rNPC1L1 cells.

Caco2 cells have been widely used as an *in vitro* model for cholesterol uptake [23]–[27]. Furthermore, cholesterol uptake by these cells can be further enhanced by exogenous expression of rNPC1L1 [28], [29]. To examine the effect of fomiroid A on NPC1L1-dependent cholesterol uptake, we used lentivirus-mediated infection to establish Caco2 cells stably expressing rNPC1L1 (Caco2/rNPC1L1). Expression of rNPC1L1 was confirmed by western-blot analysis using anti-NPC1L1 antibody in addition to the expression of endogenous hNPC1L1 in Caco2/mock cells (Fig. 5A).

[^3^H]cholesterol uptake by Caco2/rNPC1L1 cells was approximately 2-fold higher than that in Caco2/mock cells (Fig. 5B). Fomiroid A (30 µM) treatment significantly reduced cholesterol uptake in both cells, as did ezetimibe treatment. However, because cells can absorb hydrophobic cholesterol in micelles via passive diffusion, it is possible that the effects of these chemicals were obscured by NPC1L1-independent cholesterol uptake. To resolve this issue, we analyzed esterified cholesterol, the storage form of intracellular cholesterol. The formation of esterified cholesterol is suppressed in Caco2 cells subjected to NPC1L1 knockdown or ezetimibe treatment [30]–[32]. As shown in Fig. 5C, the amount of esterified cholesterol in Caco2/rNPC1L1 cells was approximately 8-fold higher than that in Caco2/mock. Fomiroid A inhibited the formation of esterified cholesterol in Caco2/mock and rNPC1L1 cells in a dose-dependent manner. In Caco2/rNPC1L1 cells, cholesterol ester formation was reduced to 29.2±3.6% by ezetimibe and to 14.4±1.2% by 30 µM fomiroid A treatment. These results suggest that fomiroid A prevents the uptake of cholesterol and the formation of esterified cholesterol by inhibiting NPC1L1, and that its inhibitory activity is as strong as that of ezetimibe.

## Discussion

We found a compound in the extract of the mushroom *Fomitopsis nigra* that directly binds to NPC1L1, a key protein involved in cholesterol absorption. We purified the active compound and named it fomiroid A. Fomiroid A strongly inhibited NPC1L1-mediated cholesterol uptake, as effectively as ezetimibe, via binding to NPC1L1.

The inhibitory effect of fomiroid A on [^3^H]EZG binding to NPC1L1 apparently showed a biphasic pattern (Fig. 2). NPC1L1 may have two different binding sites with higher and lower affinity for fomiroid A. However, because some nonspecific effects on membrane may occur at high concentrations, it is also possible that fomiroid A shows a weak allosteric effect at µM order on NPC1L1.

Fomiroid A is structurally similar to lanosterol, a precursor of cholesterol biosynthesis, which binds to the NTD in the same manner as cholesterol [15]. Therefore, we initially expected fomiroid A to bind the NTD and thereby inhibit EZG binding. However, under the conditions we examined, we did not find any effect of lanosterol on EZG binding to NPC1L1 (S3A Fig.). Similarly, 25-hydroxycholesterol, which also binds the NTD [15], [16], had no effect on EZG binding to NPC1L1 (S3B Fig.). These observations suggest that sterol binding to NTD did not affect EZG binding, and that fomiroid A exerts its inhibitory effect by binding to another site on NPC1L1.

Compounds that bind to the second sterol-binding site of NPC1L1, which is distinct from both the NTD and the ezetimibe-binding site, exhibit pharmacological chaperone activity against the NPC1L1 L1072T/L1168I mutant. Normally, this mutant localizes predominantly to the ER, but these pharmacological chaperones re-localize it to the plasma membrane [18], [19]. We examined the pharmacological chaperone activity of fomiroid A by confocal microscopy (Fig. 3A), western blotting (Fig. 3B, C), and FACS analysis (Fig. 4). Together, the results indicated that fomiroid A has pharmacological chaperone activity, and thus rescues the L1072T/L1168I mutant from the ER and re-localizes it to the plasma membrane. Ezetimibe, which binds to the second extracellular loop [12], did not exhibit such activity (S4 Fig.), as previously reported [18]. These results suggest that the primary binding site of fomiroid A is not the ezetimibe-binding site in the second extracellular loop or the cholesterol-binding site in the NTD, but rather the second sterol-binding site.

To analyze the effect of fomiroid A on cholesterol uptake, we used Caco2 cells stably expressing rNPC1L1 (Caco2/rNPC1L1). Cholesterol uptake was enhanced by 2-fold in these cells, indicating that NPC1L1 clearly contributed to cholesterol uptake (Fig. 5B). However, the inhibitory effect of ezetimibe on cholesterol uptake was modest compared to the dramatic effect on cholesterol absorption *in vivo* [3]–[5], [33], probably because cholesterol in micelles can passively diffuse into cells, as previously reported [24], [34]. To rule out the effects of passive diffusion, we analyzed the amount of esterified cholesterol. Cholesterol absorbed via NPC1L1 is transported into the ER and esterified by acyl-coenzyme A:cholesterol acyltransferase (ACAT) to yield a storage form of cholesterol. NPC1L1 knockdown in Caco2 cells markedly reduces the amount of esterified cholesterol [30]. Indeed, ezetimibe strongly inhibited the formation of cholesterol ester in both Caco2/rNPC1L1 and Caco2/mock cells (Fig. 5C). The inhibitory effect of fomiroid A was as strong as that of ezetimibe: the formation of cholesterol ester in Caco2/rNPC1L1 was inhibited by 71% and 86% by ezetimibe and fomiroid A treatment, respectively, at 30 µM. The ACAT inhibitory activity of ezetimibe is very weak [31], [35], and the ACAT inhibitory activity of fomiroid A remains to be examined. We have not succeeded in preparing enough amount of fomiroid A for animal model experiments. Effect of fomiroid A and its synergistic effect with ezetimibe on the intestinal cholesterol absorption are to be examined in the near future.

In conclusion, the results of this study show that the natural product fomiroid A, a novel compound isolated from *Fomitopsis nigra*, binds to NPC1L1, probably at the second sterol-binding site, and inhibits cholesterol uptake and cholesterol ester formation. Thus, fomiroid A may be useful as a novel inhibitor of cholesterol absorption and a molecular tool for understanding the mechanism of NPC1L1-mediated cholesterol uptake.

## Supporting Information

S1 Fig Binding of [^3^H]ezetimibe glucuronide to membranes from HEK293 cells stably expressing rNPC1L1 and HA tagged hNPC1L1. HEK293/rNPC1L1 or HA-hNPC1L1-EGFP cells were treated with 4 mM sodium butyrate for 24 h, and then membrane fractions were prepared. The membranes (37.5 µg protein) were incubated with 25 nM [^3^H]EZG for 1 h at room temperature. Nonspecific binding was measured with 100 µM ezetimibe. Radioactivity was measured on a TopCount. Values represent means ± S.E. (n = 4).
(TIF)

S2 Fig **Rescue of mislocalized HA tagged NPC1L1^L1072T/L1168I^.** HEK/HA-hNPC1L1^L1072T/L1168I^-EGFP cells were incubated in the absence or presence of 10 µM fomiroid A for 24 h, and then plasma membranes (PM) were stained with CellMask Orange. Arrows show the rescue of the mislocalized HA-L1072T/L1168I mutant by fomiroid A treatment. Scale bar, 10 µm.
(TIF)

S3 Fig Effect of lanosterol or 25-hydroxycholesterol on the interaction between [^3^H]ezetimibe-glucuronide and NPC1L1. A–B, Membranes (37.5 µg protein) from HEK293/rNPC1L1 were incubated for 1 h at room temperature with 25 nM [^3^H]EZG in the presence of increasing concentrations of unlabeled lanosterol (A) or 25-hydroxytyrosol (B). Nonspecific binding was measured with 100 µM ezetimibe.
(TIF)

S4 Fig **Effect of ezetimibe on cell surface expression of HA-tagged NPC1L1^L1072T/L1168I^.** HEK/HA-hNPC1L1^L1072T/L1168I^-EGFP cells were incubated in the absence or presence of 10 µM ezetimibe for 24 h. Cell-surface expression of HA-L1072T/L1168I mutant was detected by staining with anti-HA and Alexa Fluor 633–conjugated anti–mouse IgG antibodies, and then quantitated by FACS analysis. The percentages in the upper right regions of the panels indicate the proportion of cells double-positive for anti-HA antibody and EGFP.
(TIF)
