# Supplementary figures and images for: Fomiroid A, a Novel Compound from the Mushroom Fomitopsis nigra, Inhibits NPC1L1-Mediated Cholesterol Uptake via a Mode of Action Distinct from That of Ezetimibe
Source: PLoS One. 2014 Dec 31;9(12):e116162. doi: 10.1371/journal.pone.0116162 (PMC4281142; doi:10.1371/journal.pone.0116162)

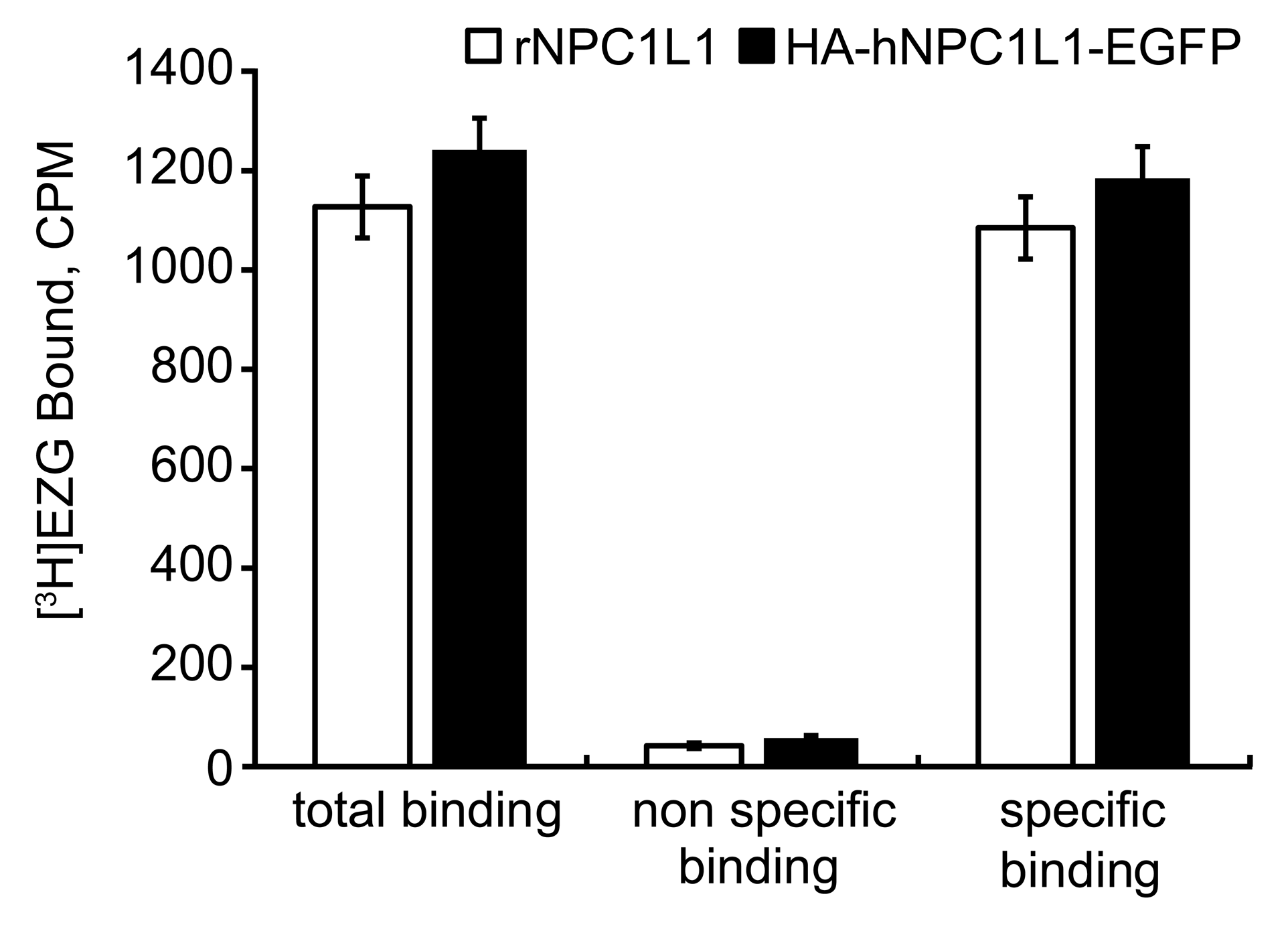

Supplement: S1 Fig — Binding of [3H]ezetimibe glucuronide to membranes from HEK293 cells stably expressing rNPC1L1 and HA tagged hNPC1L1. HEK293/rNPC1L1 or HA-hNPC1L1-EGFP cells were treated with 4 mM sodium butyrate for 24 h, and then membrane fractions were prepared. The membranes (37.5 µg protein) were incubated with 25 nM [3H]EZG for 1 h at room temperature. Nonspecific binding was measured with 100 µM ezetimibe. Radioactivity was measured on a TopCount. Values represent means ± S.E. (n = 4). (TIF) [file pone.0116162.s001.tif]

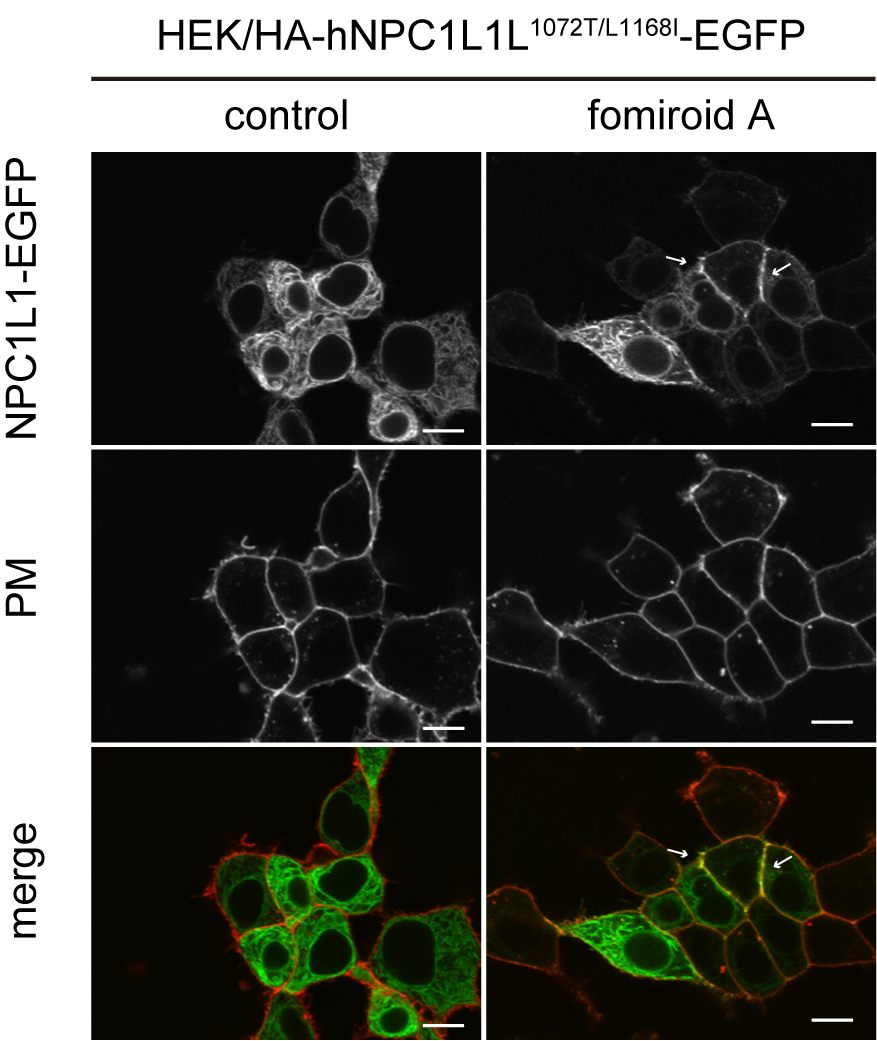

Supplement: S2 Fig — Rescue of mislocalized HA tagged NPC1L1L1072T/L1168I. HEK/HA-hNPC1L1L1072T/L1168I-EGFP cells were incubated in the absence or presence of 10 µM fomiroid A for 24 h, and then plasma membranes (PM) were stained with CellMask Orange. Arrows show the rescue of the mislocalized HA-L1072T/L1168I mutant by fomiroid A treatment. Scale bar, 10 µm. (TIF) [file pone.0116162.s002.tif]

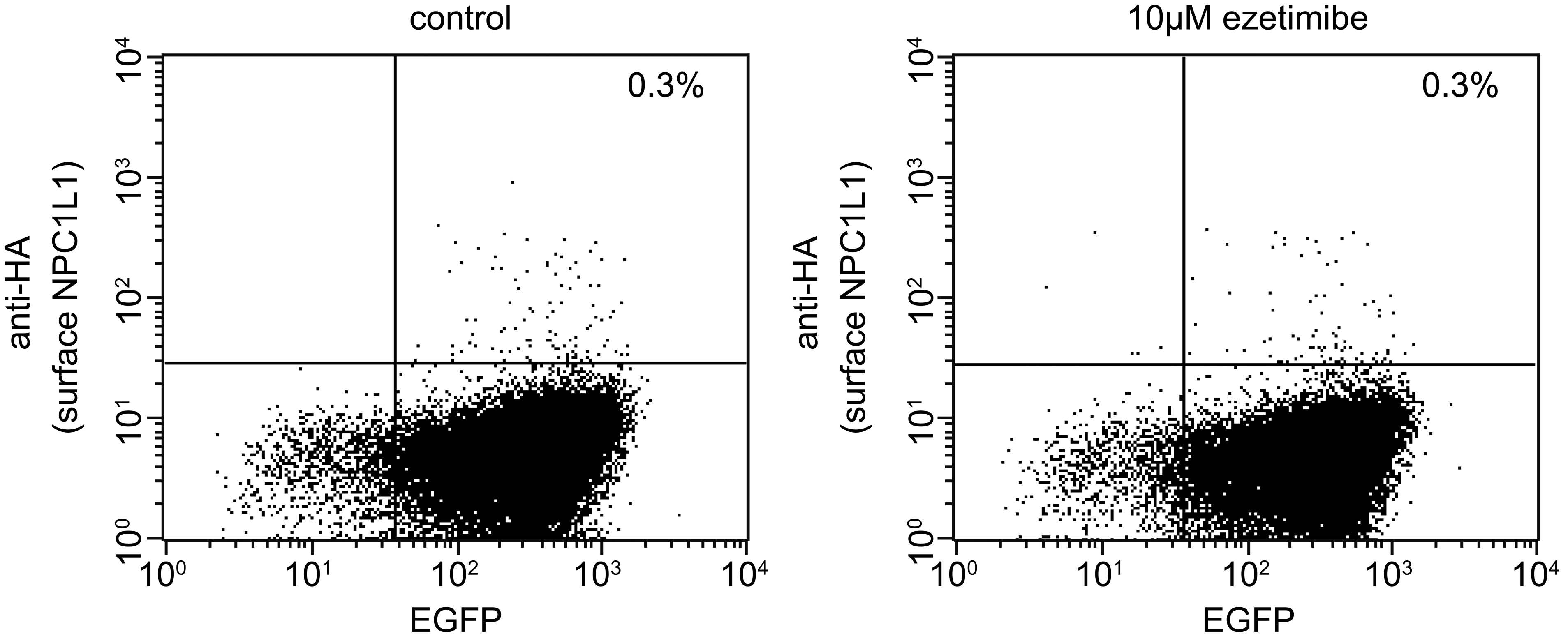

Supplement: S4 Fig — Effect of ezetimibe on cell surface expression of HA-tagged NPC1L1L1072T/L1168I. HEK/HA-hNPC1L1L1072T/L1168I-EGFP cells were incubated in the absence or presence of 10 µM ezetimibe for 24 h. Cell-surface expression of HA-L1072T/L1168I mutant was detected by staining with anti-HA and Alexa Fluor 633–conjugated anti–mouse IgG antibodies, and then quantitated by FACS analysis. The percentages in the upper right regions of the panels indicate the proportion of cells double-positive for anti-HA antibody and EGFP. (TIF) [file pone.0116162.s004.tif]
